# Paediatricians’ awareness on orthodontic problems and related conditions—a national survey

**DOI:** 10.1186/s40510-019-0285-x

**Published:** 2019-08-19

**Authors:** Marianna Koufatzidou, Despina Koletsi, Eirini Iouliani Basdeki, Nikolaos Pandis, Argy Polychronopoulou

**Affiliations:** 1 School of Dentistry, National and Kapodistrian University of Athens, Athens, Greece; 2 Clinic of Orthodontics and Paediatric Dentistry, Center of Dental Medicine, University of Zurich, Zurich, Switzerland; 3 Department of Orthodontics and Dentofacial Orthopedics, Dental School/Medical Faculty, University of Bern, Bern, Switzerland; 4 Department of Preventive and Community Dentistry, School of Dentistry, National and Kapodistrian University of Athens, Athens, Greece

**Keywords:** Orthodontic knowledge paediatricians, Reference to orthodontists, Paediatricians’ role, Oral health, Orthodontic principals, Reference, Education, Paediatric residency

## Abstract

**Background:**

Correction of dentofacial deformities via orthodontics is an integral part of oral health as promotes optimal function, periodontal health, aesthetics and overall oral health-related quality of life. The aim of this study was to examine whether paediatricians refer their patients to orthodontists, whether they have sufficient knowledge in basic orthodontic principles and whether they examine their patients for orthodontic abnormalities.

**Results:**

We conducted a survey study of paediatricians in Greece. Questionnaires were completed by 96 out of 123 paediatricians (response rate 78%). In the assessment of the examination of the oral cavity, a low frequency of examination of the position of the teeth (54%) and jaws (51%) was found. Reasons paediatricians referred patients to specialists varied from mouth breathing-snoring 24% (23/96) to face or teeth asymmetry 87% (84/96). In the multivariable analyses for the effect of gender, work sector or years of experience in the decision for orthodontic referral, we could not identify any significant predictors.

**Conclusions:**

The results of this study indicate that there was variability regarding orthodontic knowledge among paediatricians. Although the majority were aware of the importance of examination of the oral cavity, they did not have the appropriate knowledge to perform a full and systematic screening for orthodontic problems. The probability of referral was different for the various orthodontic anomalies.

## Background

Correction of dentofacial deformities via orthodontics is an integral part of oral health as it promotes optimal function, periodontal health, aesthetics, and overall oral health-related quality of life [1, 2].

Inadequate lip coverage, increased overjet with labial proclination of maxillary incisors, and anterior open bite are examples of dentofacial deformities that may be successfully managed with orthodontic treatment, resulting in functional improvement and reduction in the risk of maxillary incisor and gingival tissue trauma [1–5]. Dentofacial abnormalities have been associated with speech disorders [3] and people that have undergone orthodontic treatment are able to clean their teeth more effectively, which may result to a significant reduction in dental caries and periodontal disease [5].

Paediatricians are responsible for the health status of infants and children and as such, oral health cannot be excluded from the overall health assessment [7]. Furthermore, there is evidence that young children are more likely to visit a medical office than a dental one [8, 9] Therefore, it is important that paediatricians conduct initial orthodontic screenings in order to diagnose abnormalities early and refer the patients accordingly.

Paediatricians may help in early diagnosis of orthodontic problems and this may improve the treatment outcome and its stability over the years [10, 11]. For instance, unilateral posterior crossbite has been documented as one of the most frequent malocclusions of the primary teeth of Caucasian children. If left untreated or not timely treated, lateral mandibular displacement may lead to facial asymmetry due to undesirable growth modification [10]. It is therefore important to treat crossbites during the early mixed dentition in order to establish a physiologic transverse occlusion in addition to a skeletal equilibrium [11]. Lastly, obese as well as allergic children may develop mouth breathing and obstructive sleep apneas which are associated with types of malocclusion that need an early diagnosis and specific treatment [12]. In addition to the correction of a functional abnormality, orthodontic treatment at an early age may provide patients with a good aesthetic outcome which can have positive consequences on their self-esteem [3, 5, 6].

Although paediatricians are expected to be knowledgeable about oral health-related issues in order to fulfil their responsibilities as professionals, the educational curriculum of paediatric specialty rarely includes oral health education and when it does, the devoted time is limited. [7, 15].

The study hypothesis was that paediatricians might not have sufficient knowledge to examine their patients for orthodontic-related conditions and might not refer them to an adequate level to the orthodontists. The aim of this study was to examine whether paediatricians examine their patients as far as orthodontic problems are concerned, whether they have sufficient knowledge in basic orthodontic principles and whether they refer their patients for orthodontic problems.

## Methods

The study was not a priori registered. Informed consent was waivered due to the anonymous and voluntary character of this survey.

### Sample

A questionnaire was handed to paediatricians who participated in the 55th Panhellenic Congress for Pediatrics, which was held in Kos from the 2nd until the 4th of June 2017. Paediatricians from all over Greece were asked to answer the survey that was given to them.

### Survey

The questionnaire consisted of four parts. First, demographic characteristics such us age, gender, and work sector, were recorded. On the second part, the participants were queried about their examination ritual, whether they examine the oral cavity, the position of teeth and jaws and on their knowledge in specific orthodontic anomalies such as crowding, overjet, and the prevalence in their patients. On the third part, their referral practices to orthodontists and their reasoning were assessed. Finally, their personal orthodontic experience and the source of their orthodontic education, if any, was recorded.

### Survey administration

The survey was handed to study participants in “paper and pencil” format. The purpose of the project was communicated to all the participants and anonymity was ensured. The administration of the survey and the data collection procedures were conducted by two—undergraduate students of the School of Dentistry, National and Kapodistrian University of Athens—co-authors of this manuscript.

### Statistical analysis/analytical approach

Descriptive statistics were performed for responders’ demographic data. To test the association between paediatricians’ related characteristics and overall referral to orthodontists or otherwise, Pearson chi-square test and Fisher’s exact test were undertaken as appropriate. Cross-tabulations and frequency distributions were presented for the examination of the oral cavity and orthodontic-related problems, or reasons for referral to a specialist. Univariable and multivariable logistic regression analyses were performed to assess the effect of gender, work sector or years of experience in the decision for orthodontic referral. The variables gender, work sector, and years of experience served as the independent variables, while the decision for orthodontic referral was the dependent or outcome variable. Model fit was checked using the Hosmer–Lemeshow test. The level of significance was pre-specified at *p* < 0.05.

All statistical analyses were conducted with Stata version 15.1 software (Stata Corporation, College Station, TX, USA).

## Results

A total of 96 out of 123 paediatricians returned the questionnaires completed (response rate 78%). The completion response frequency for demographic variables ranged from 80 to 99 percent, with age bearing the lowest fraction of questionnaire completion. Demographic characteristics are available in Table 1. Female responders (64/95, 67%) predominated male responders, while the mean age was 45.2 years old (SD 13.0). Fifty-five percent reported working in the private sector (51/93), while only 21% (18/85) reported having obtained a subspecialty. Most of the responders reported working duration times up to 50 h per week (53/92, 58%).

**Table 1:** Demographic characteristics of paediatricians by referral pattern to orthodontist

	Referral to orthodontist						
	No		Yes		Total		*p* value
	*N*	%	No.	%	*N*	%	
Gender							0.43*
Male	23	74	8	26	31	100	
Female	52	81	12	19	64	100	
Total	75	79	20	21	95	100	
Age							0.01^#^
26–35	22	100	0	0	22	100	
36–45	14	74	5	26	19	100	
46–55	10	63	6	37	16	100	
over 55	15	79	4	21	19	100	
Total	61	80	15	20	76	100	
Subspecialty							0.35*
No	55	82	12	18	67	100	
Yes	13	72	5	28	18	100	
Total	68	80	17	20	85	100	
Work sector							0.02^#^
Public	33	92	3	8	36	100	
Private	34	67	17	33	51	100	
Both	5	83	1	17	6	100	
Total	72	77	21	23	93	100	
Years at work							0.05^#^
1 to 5	21	95	1	5	22	100	
6 to 15	18	82	4	18	22	100	
16 to 30	19	66	10	34	29	100	
Over 30	8	73	3	27	11	100	
Total	66	79	18	21	84	100	
Hours per week							0.55^#^
1 to 25	7	70	3	30	10	100	
26 to 50	32	74	11	26	43	100	
51 to 75	20	77	6	23	26	100	
over 75	12	92	1	8	13	100	
Total	71	77	21	23	92	100	
Patients per day							0.68^#^
1 to 10	24	73	9	27	33	100	
11 to 20	28	82	6	18	34	100	
21 to 30	10	77	3	23	13	100	
over 30	9	90	1	10	10	100	
Total	71	79	19	21	90	100	

*Pearson chi-squared test

^#^Fisher’s exact test

Assessment of the examination of the oral cavity is presented in Table 2. Although paediatricians examined the mucosa (95/96, 99%), the tongue (93/96, 97%) and even the teeth of their patients (83/96, 86%), the examination of the position of the teeth (52/96, 54%) and jaws (49/96, 51%) was rarely performed.

**Table 2:** Responses of participants in relation to examination of the oral cavity

	Examination of oral cavity elements		
	No	Yes	Total
	*N* (%)	*N* (%)	*N* (%)
Oral cavity	0 (0)	96 (100)	96 (100)
Mucosa	1 (1)	95 (99)	96 (100)
Tongue	3 (3)	93 (97)	96 (100)
Teeth	13 (14)	83 (86)	96 (100)
Teeth position	44 (46)	52 (54)	96 (100)
Jaw position	47 (49)	49 (51)	96 (100)

Regarding paediatricians’ awareness of the prevalence of common orthodontic anomalies in their patients, their responses varied from 31 to 95%. Thirty out of 96 (31%) examined their patients for crossbite, 34 out of 96 (35%) for overbite, while 84 out of 96 (87%) for prognathism, and 91 out of 96 (95%) for paranormal functional habits like finger sucking. (Table 3).

**Table 3:** Responses of paediatricians in relation to examination of the orthodontic problems

	Examination of orthodontic-related elements		
	No	Yes	Total
	*N* (%)	*N* (%)	*N* (%)
Crowding	42 (44)	54 (56)	96 (100)
Crossbite	66 (69)	30 (31)	96 (100)
Overbite	62 (65)	34 (35)	96 (100)
Missing teeth	39 (41)	57 (59)	96 (100)
Spaces	41 (43)	55 (57)	96 (100)
Prognathism	12 (13)	84 (87)	96 (100)
Retrognathism	39 (41)	57 (59)	96 (100)
Habits (i.e., finger-sucking)	5 (5)	91 (95)	96 (100)

Their responses for the reasons for referral to specialists differed for each condition from 24% (23/96) for mouth breathing-snoring to 87% (84/96) for face or teeth asymmetry. More specifically, 36% (35/96) tended to refer for delayed eruption, 73% (70/96) for jaw deviation, 56% (54/96) for crowding, 35% (34/96) for crossbite, 39% (37/96) for overbite, 49% (47/96) for spaces, 79% (76/96) for prognathism, 58% (56/96) for retrognathism, and 26% (25/96) for delayed teeth change (Table 4).

**Table 4:** Responses of paediatricians in relation to the reason for orthodontic referral

	Referral related		
	No	Yes	Total
	*N* (%)	*N* (%)	*N* (%)
Early tooth loss	47 (49)	49 (51)	96 (100)
Delayed eruption	61 (64)	35 (36)	96 (100)
Difficulty in biting	49 (51)	47 (49)	96 (100)
Sounds from tmj	55 (57)	41 (43)	96 (100)
Face/teeth asymmetry	12 (13)	84 (87)	96 (100)
Jaw deviation (mouth closing)	26 (27)	70 (73)	96 (100)
Mouth breathing/snoring	73 (76)	23 (24)	96 (100)
Crowding	42 (44)	54 (56)	96 (100)
Crossbite	62 (65)	34 (35)	96 (100)
Overbite	59 (61)	37 (39)	96 (100)
Missing teeth	51 (53)	45 (47)	96 (100)
Grinding at sleep	60 (63)	36 (37)	96 (100)
Spaces	49 (51)	47 (49)	96 (100)
Prognathism	20 (21)	76 (79)	96 (100)
Retrognathism	40 (42)	56 (58)	96 (100)
Delayed teeth change	71 (74)	25 (26)	96 (100)

*tmj*, temporomandibular joint

We examined the paediatricians’ referral patterns to orthodontists in relation to the demographic characteristics such as gender, age, work sector, subspecialty, and years of work. In the univariable logistic regression, there was a statistically significant result for the effect of the work sector. Notwithstanding, in the multivariable regression model for the effect of gender, work sector or years of experience in the decision for orthodontic referral, we could not identify any significant predictors overall. More specifically, we could only detect a 12.75 fold increase in the odds for referral for those with 21 to 30 years of working experience compared to those with 0–10 years in practice, after adjusting for gender and work sector (OR = 12.75, 95% CIs 1.16, 140.26; *p* value = 0.04). However, there was great uncertainty in the estimate (Table 5).

**Table 5:** Univariable and multivariable logistic regression for the effect of sex, work sector, and years of experience (as a proxy measure of age and years at work) on orthodontic referrals (*n* = 90)

Category	Univariable			Multivariable		
	OR	95% CI	*p* value	OR	95% CI	*p* value
Gender						
Male	Reference					
Female	0.66	0.24, 1.84	0.43	1.01	0.29, 3.49	0.98
Work sector			0.04*			0.60*
Public	Reference					
Private	5.50	1.47, 20.54	0.01	1.96	0.40, 9.51	0.41
Both	2.20	0.19, 25.52	0.53	0.87	0.06, 12.00	0.92
Years of experience			0.10*			0.16*
0–10	Reference					
11–20	9.33	1.04, 84.09	0.05	6.70	0.57, 78.50	0.13
21–30	16.00	1.82, 140.92	0.01	12.75	1.16, 140.26	0.04
Over 30	8.00	0.82, 78.47	0.07	4.81	0.34, 67.49	0.24

*Wald test for the overall effect

## Discussion

The variability of the orthodontic examination practices and possibly the ability to recognize the prevalence of orthodontic problems is reflected in the patient referral patterns from the paediatrician to the orthodontist. While conditions such as face asymmetry and prognathism were readily recognized and resulted in high referral frequencies, other anomalies were not common reasons for referrals. Orthodontic problems less likely to result in referrals included mouth breathing—snoring, delayed eruption, crossbite, overbite, and nocturnal grinding.

The lack of orthodontic prevention and screening, at an early age, is manifested throughout the bibliography. In the Albanian population, 85% have been reported to present oral habits like pacifier sucking, while a severe and very severe need for orthodontic treatment was found in up to 17% [13, 14]. Studies in other counties, namely, in Austria and Croatia, manifest a great need for orthodontic treatment among children aged 8–10 and adolescents aged 12–18, respectively. It is therefore obvious that orthodontic prevention, at an early age if possible, should be reinforced [15, 16].

We could not identify any other survey regarding orthodontic screening and referral from paediatricians in the literature. Therefore, we could only compare our results with studies assessing paediatrician’s knowledge and referrals for oral hygiene and dental caries [17–19]. In a national survey with 1618 post-residency members of the American Academy of Pediatrics, 90% of the responders claimed that they should examine the oral cavity and teeth, while only 54% claimed to examine the teeth of half of their 0–3-year-old patients. Lack of training was the most common reason for not performing an oral examination [17]. In another study including general dentists, paediatric dentists, and paediatricians, only 5% of the paediatricians recommended a dental visit by the age of 1 year old [18]. Therefore, it may be presumed that paediatricians have limited to basic dental education which leads to low confidence for oral cavity screening and recommendations or consultation [17, 18].

Ideally, orthodontic screenings should be performed to all children in both dental and paediatric practices as each specialty can provide care and advice for their patients’ orthodontic health. Paediatricians built a relationship with both patients and parents from an early age. As they usually examine their patients before orthodontists do, they have the chance to advise, guide, and refer as deemed necessary.

One limitation of this study is the relatively small sample size which included 96 participants. There was no formal assessment on how responders and non-responders might have differed. Some of the non-responders might have had different answers than those of the responders and this might have had an implication for the generalizability of the study findings. The origin of the participants of this study was not assessed. Therefore, we could not identify inter-area differences. Responder bias is a common problem in studies involving questionnaires. Some of the participants may have answered more favorably regarding their examination rituals in order to appear more comprehensive in their examination than what they actually do in practice. This is a study reflecting on the attitude of the participants on a specific time and may differ in general. Recall bias may be present in this study, since questionnaire-studies retrieve their results from participants who have to recall their experiences and knowledge in order to answer.

Lastly, as there was no related previous study on the topic, we could not have used a pre- existing survey/questionnaire as a validated guide. Thus, the questionnaire used was custom- made and formal data about its validity are lacking.

## Conclusions

The results of this study indicate that there is a great variability regarding orthodontic problems and examination practices among paediatricians. Although most of the practitioners are aware of the need for examining the oral cavity, they do not seem to undertake a systematic orthodontic.

There is a need for the two specialties to work together for the benefit of the patient. A possible solution, in order to establish effective cooperation, is through the inclusion of dental courses regarding orthodontics in paediatric residency curriculum and through inter-professional seminars and interaction.

## Data Availability

The datasets used and/or analysed during the current study are available from the corresponding author on reasonable request.

## Abbreviations

CIs: Confidence intervals
OR: Odds ratio
SD: Standard deviation
